# Relationship between Firefighter Physical Fitness and Special Ability Performance: Predictive Research Based on Machine Learning Algorithms

**DOI:** 10.3390/ijerph17207689

**Published:** 2020-10-21

**Authors:** Datao Xu, Yang Song, Yao Meng, Bíró István, Yaodong Gu

**Affiliations:** 1 Faculty of Sports Science, Ningbo University, Ningbo 315211, China; xudatao3@gmail.com (D.X.); nbusongyang@hotmail.com (Y.S.); jymengyao@gmail.com (Y.M.); 2 Doctoral School of Safety and Security Sciences, Obuda University, 1034 Budapest, Hungary; biro-i@mk.u-szeged.hu; 3 Faculty of Engineering, University of Szeged, 6724 Szeged, Hungary

**Keywords:** tactical population, partial least-squares regression (PLSR), linear regression predictive analysis

## Abstract

Firefighters require a high level of physical fitness to meet the demands of their job. The correlations and contributions of individual physical health parameters to the tasks of firefighting would enable firefighters to focus on the effects of specific physical conditions during their physical training programs. Therefore, the purpose of the present study was to identify the relationships between various physical health parameters (weight, maximum oxygen uptake, body fat percentage, upper body muscular power and lower body muscular power) and performance on simulated firefighting ability tasks, which included a set of seven tasks (rope climb, run 200 m round trip with load, 60 m carrying a ladder, climb stairs with load, evacuation of 400 m with supplies, run 5 km with an air respirator, run 100 m with the water hose). Through use of a partial least-squares regression (PLSR) algorithm to analyze the linear correlation, we revealed the change in various training performances of specific ability tests with physical fitness parameters. The present study demonstrated significant relationships among physical health parameters and performance on simulated firefighting ability tasks, which also represent that those parameters contributed significantly to the model’s predictive power and were suitable predictors of the simulated firefighting tasks score.

## 1. Introduction

The past few decades have seen the construction of a large number of high-rise buildings and large commercial centers, which have placed greater demands on the skills and physical abilities of firefighters [1,2]. Therefore, this places a high demand on the physical ability and health of firefighters. Firefighters are required to wear a heat suit respirator that is carried on their backs. The weight of the self-contained working tools used by firefighters is also additional. Both the total weight of the thermal suite, which may be 25 kg, and the increased inhalation resistance at the scene of the fire and when using the respirator place extremely high demands on the firefighter’s physical capabilities [1,3]. Furthermore, firefighters engage in heavy muscle work, they must climb stairs and ladders, carry and use heavy tools, usually on their heads or in awkward positions where they may be called upon to perform difficult rescue operations [4].

Numerous studies have shown that firefighters need a higher level of physical fitness to meet the demands of their profession [5,6,7,8]. Firefighting requires a high level of aerobic and anaerobic fitness as well as muscular strength, endurance, explosive power, reaction time, and so on. Albert et al. [9] found that suboptimal physical fitness and excess weight lead to a mismatch with the high energy demands required of firefighters, a combination that may trigger sudden on-duty cardiovascular events. The physiological demands of firefighters during simulated fire suppression have been measured in several studies. Heart rate (HR) has been determined to be up to 90% of the HR maximum [10,11,12,13]. At the same time, numerous studies have also demonstrated that the maximum oxygen uptake of firefighters is least 40 mL/kg/min [14,15,16]. However, performance in a general aerobic fitness test is only marginally related to performance in rescue operations or firefighting activities. Mamen et al. [17,18,19] found that the peak O_2_ uptake (VO_2_) was approximately 45 mL/kg/min, measured by Norwegian Labour Inspection Authority (NLIA) and a Canadian test. The Canadian test had lower perceived strain scores than the Norwegian test and shorter exercise times at high VO_2_.

Firefighting is a very dangerous profession and requires a great deal of physical fitness to pass the initial screening test. Firefighters have been reported to be at increased risk of cardiovascular disease (CVD) if they have a higher BMI, which is associated with an increased risk of CVD [20]. In addition, among firefighters, high BMI was also associated with a higher risk of musculoskeletal injury [21]. Improvements in cardiopulmonary function may have a positive effect. With the impact on cardiovascular health and overall mortality, small changes in BMI may be associated with a reduction in myocardial metabolic risk factors [22,23,24]. Therefore, it is important to assess the relationship between body composition and fitness in firefighters, especially among military firefighters for which scientific data are lacking.

Strength training is also an essential part of a firefighter’s career. Nazari et al. [25] found that, compared to the general population, firefighters had about the same aerobic capacity but a much higher level of physical strength. As firefighters age, their aerobic capacity declines, but their upper and lower limb strength levels remain the same. According to the report, upper body strength shows different manifestations in different professions. Compared with firefighters, male police officers are younger, heavier, have higher body fat rates, higher rates of obesity and greater upper body strength [26]. Compared with firefighters, policewomen are younger, lighter, thinner and have less upper body strength. Kleinberg et al. [27] found that the size and mass of lower limb muscles are important contributors to critical fire tasks, and that resistance training has been shown to improve firefighting tasks. Giuliani et al. [28] also found that lower limb muscle strength was positively correlated with fatigue recovery.

Previous research has extensively explored firefighter health and fitness, but most studies have focused only on the influence of the individual firefighter’s physical fitness (such as body fat percentage, cardiopulmonary function, upper and lower limb strength) on specific ability performance [14,15,17,18,28]. There has been no exploration of the correlation between firefighter fitness indicators and regular training performance. Because there are multiple correlations between predictor variables (firefighter physical fitness) and response variables (firefighter special ability performance), partial least-squares regression (PLSR) was selected for regression analysis in this study.

The primary purpose of this study was to identify the relationships between the physical health parameters and various training performances of firefighters in specific ability tests. We used a partial least-squares regression (PLSR) algorithm to linearly correlate physical fitness parameters with changes in various performances of specific ability tests. Through the establishment of a multiple regression PLSR algorithm, a linear model between physical health parameters and various training performance of firefighters in specific ability tests was trained, verified and tested to provide scientific reference for firefighters’ physical training.

## 2. Materials and Methods

### 2.1. Participants

A total of 20 firefighters (age: 25.65 ± 2.97 years; height: 172.4 ± 4.8 cm; mass: 69.0 ± 8.9 kg) from southeast China took the physical fitness test. All subjects had no history of injury for six months. Subjects were informed of the risks of the experiment and signed an informed consent form before testing. The survey was approved by the Institutional Review Board of Ningbo University for the use of human subjects. The testing sessions took place in May. The average temperature and humidity during the sessions were 25 °C and 50%, respectively.

### 2.2. Test Protocol and Data Collection

The study was divided into two phases. Firstly, firefighters performed the ability test. Secondly, they underwent fitness assessments approximately 2 weeks later. The second phases were designed to provide information about firefighters’ test score and physical fitness, respectively. Efforts to improve training and physical fitness of firefighters included investigation of relationships between various fitness parameters and ability tests. During the ability test, to simulate real-world gear demands, all firefighters wore protective gear with a total weight of 20–25 kg. All physical and health fitness tests were administered by experienced testers with the assistance of firefighter instructors who were trained in the proper techniques, formats and procedures for all tests.

#### 2.2.1. Ability Test

The test was administered to all firefighters by the same trained instructor. The test included rope climb (Y1), run 200 m round trip with load (Y2), run 60 m carrying a ladder (Y3), climb stairs with load (Y4), evacuation of 400 m with supplies (Y5), run 5 km with an air respirator (Y6) and run 100 m with a water hose (Y7). The test is described as follows:

Y1: Firefighters were asked to use ropes through 12–16 successive upper and lower limb coordination movements to climb a four-story building (about 15 m). The movements are as follows: hanging from the rope, lifting the knees to grasp the rope, stepping, extending the hips and bending the arms, and standing grasp. This is done until the designated height is reached. Y2: Carry a 30 kg weight as fast as possible over a distance of 50 m, and repeat this four times. Y3: Carry a ladder during a 60 m sprint, which weighs 10.32 kg and is 3.49 m long and 0.63 m wide. Y4: Climb from the first-floor entrance to the 10th floor with a 35 kg load fire apparatus, each floor being 3.06 m. Y5: Mark a starting line on a 400 m loop training field and the equipment lines at 100, 200 and 300 m from the starting line. On the starting line, there are two foam buckets with a weight of 16 kg each; on the 100 m equipment line there is one truck tire with a weight of 40 kg; on the 200 m equipment line there is one Liquefied Petroleum Gas (LPG) bottle with a weight of 15 kg; on the 300 m equipment line there is one dummy with a weight of 60 kg. Y6: Subjects carried 20–25 kg air respirator apparatus on their backs and ran as fast as they could for 5 km on the 400 m loop of the training ground. Y7: Subjects put on firefighter’s gear weighing 20–25 kg and held a 40 cm diameter hose weighing 4 kg in each hand. After standing at the starting point of the 100 m obstacle course, the trainer gave the command “Start”, and the participant sprinted from the starting point to the finish line.

#### 2.2.2. Fitness Assessments

The individual test methods are described below. Once in the room, the firemen were told to sit comfortably for ten minutes. Ten minutes later, the test began. The test included maximum oxygen uptake or VO_2max_ (X2), body fat percentage (X3), upper body muscular power (X4) and lower body muscular power (X5). The test is described as follows:

X2: To test the fireman’s VO_2max_, a detailed progressive exercise test was performed with the cycle ergometer (Monark 928E, Vansbro, Sweden). Heart rate (Polar Electro RS 400, Kempe, Finland) and gas exchange data (Cortex Biophysik GmbH Metalyzer 3B, Leipzig, Germany) were continuously gathered. Firefighters warmed up at a speed of their choice for 5 min and then began the ride at 30 W, maintaining 90 rpm for the entire ride. The increase in workload for each stage was 15 W. The trial was terminated when peak VO_2max_ was reached or when the standard criteria of will fatigue were met. X3: Whole-body bioelectrical impedance was monitored using an InBody composition analyzer (InBody720, Seoul, Korea). Firefighters needed to follow the following guidelines before testing: (1) no food or drink the morning of the test, (2) no vigorous exercise in the 12 h before the test, (3) no alcohol within 48 h of the test, and (4) empty the bladder 30 min before the test. Each firefighter was asked to stand barefoot on the contact electrode of the analyzer, and when the screen showed them picking up the electrode, the subjects quickly picked up the electrode and held their hands away from the body with their palms. X4: A chest press was used to measure peak upper body power using a standard Keiser pneumatic resistance training device (Keiser Co., Ltd., Fresno, CA, USA). Measurements of upper body peak power were recorded and used for analysis at peak power. X5: The sitting leg power was used to measure the peak power of the lower body, which was the same as the upper body test and used the same equipment training system.

### 2.3. Partial Least-Squares Regression (PLSR) Prediction Model

PLSR provides a many-to-many linear regression modeling method, especially when (1) there are a lot of variables in the two groups, (2) there are multiple correlations and/or (3) the number of observation data (sample size) is relatively small. The model established by PLSR has some advantages that the traditional classical regression analysis method does not have [29]. In our study, there were multiple correlations between predictor variables (firefighter physical fitness) and response variables (firefighter special ability performance), there were many variables in the two groups (predictor variables: 5, response variables: 7), and the sample size (*n* = 20) was relatively small. Therefore, given the consistency between our study and the application conditions of PLSR analysis, PLSR was selected for regression analysis in this study.

Partial least-squares regression (PLSR) [29,30,31] was used to model the relationship between five predictors (including various physical fitness indicators of firefighters) and seven responses (including scores of specific skill assessment of firefighters). The predictive variables included weight (X1), maximum oxygen uptake or VO_2max_ (X2), body fat percentage (X3), upper body muscular power (X4) and lower body muscular power (X5). The response variables included rope climb (Y1), run 200 m round trip with load (Y2), run 60 m carrying a ladder (Y3), climb stairs with load (Y4), evacuation of 400 m with supplies (Y5), run 5 km with an air respirator (Y6) and run 100 m with a water hose (Y7).

The predictive variables and response variables were put into MATLAB (MATLAB R2019a, MASS, Natick, MA, United States) and ran with the self-compiled PLSR algorithm. The basic idea of this algorithm is to propose the first component t1 in the set of predictor variables (t1 is a linear combination of predictor variables X1, X2, …, X5). At the same time, the first component u1 is also extracted from the set of responses variables, and the correlation between t1 and u1 is required to reach the maximum degree. The regression model formulas of the prediction matrix set (X) and the reaction matrix set (Y) are as follows:

(1) $$E_{0}=\hat{t}\alpha_{1}^{T}+E_{1}$$

(2) $$F_{0}={\hat{\mu }}_{1}\beta_{1}^{T}+F_{1}$$

α_1_ = (α_11_, …, α_1m_)^T^ and β_1_ = (β_11_, …, β_1p_)^T^ are parameter vectors in regression models of prediction set and reaction set respectively, m is the total number of predictive factors, p is the total number of reaction factors, E_1_ and F_1_ are the residual matrices. The least-squares estimates of the regression coefficient vector α_1_ and β_1_ are as follows:

(3) $$\alpha_{1}=E_{0}^{T}{\hat{t}}_{1}/{\left\|{\hat{t}}_{1}\right\|}^{2}$$

(4) $$\beta_{1}=F_{0}^{T}{\hat{t}}_{1}/{\left\|{\hat{t}}_{1}\right\|}^{2}$$

Then, the residual matrixes E_1_ and F_1_ are used to replace E_0_ and F_0_ for repeated iterative operations. In this regression model, the number l of principal components to be extracted for modeling is determined by cross-validity test. Therefore, h principal components are extracted, and y_j_ is the j-th dependent variable. The squared sum of the prediction error is shown in the following equation:

(5) $$\mathrm{PRESS}\left(h\right)=\sum_{i=1}^{p}\mathrm{PRES}S_{j}\left(h\right)$$

The error squared sum of the dependent variable set Y is

(6) $$SS\left(h\right)=\sum_{j=1}^{p}SS_{j}\left(h\right)$$

When PRESS (h) reaches the minimum value, the corresponding h is the number of components sought. Usually, PRESS (h) is greater than SS (h), while SS (h) is less than SS (h − 1). Therefore, the smaller PRESS (h)/SS (h − 1) is the better. Generally, the limit value can be set as 0.05 [29,32]:

(7) $$Q_{h}^{2}=1-\mathrm{PRESS}\left(h\right)/SS\left(h-1\right)=1-0.95^{2}=0.0975$$

Therefore, when crossing validity Q_h_^2^ < 0.0975 is defined in this algorithm, the model meets the precision requirement and the extraction of components can be stopped.

The PLSR machine learning algorithm model used a data set with 80% sample size as a training set and 20% sample size as a test set. After cross-checking the training set of the model, the new data set was used to verify the model. The maximum X_max_, minimum X_min_, the difference between the maximum and minimum X_dif_ (X_max_ − X_min_) and average X_ave_ of each predictive variable were taken out (Table 1). The incremental perturbation action of a predictor variable was taken with X_min_ − 20% X_dif_, X_min_ + 0% X_dif_ (X_min_), X_min_ + 20% X_dif_, X_min_ + 40% X_dif_, X_min_ + 60% X_dif_, X_min_ + 80% X_dif_, X_min_ + 100% X_dif_ (X_max_), X_min_ + 120% X_dif_ (Table 2). A predictor variable was taken as the increment, and other predictor variables were substituted into the PLSR equation with X_ave_ to evaluate the changes in the set of response variables (special skill assessment scores).

## 3. Results

**Figure 1:**
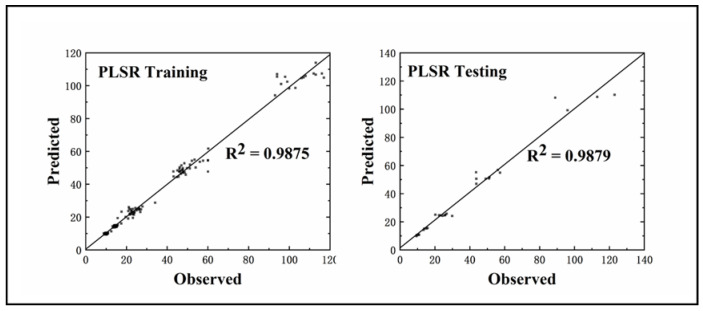
Training (**left**) and testing (**right**) accuracy of special skills assessment results of observed and predicted from the PLSR model in the firefighters.

**Figure 2:**
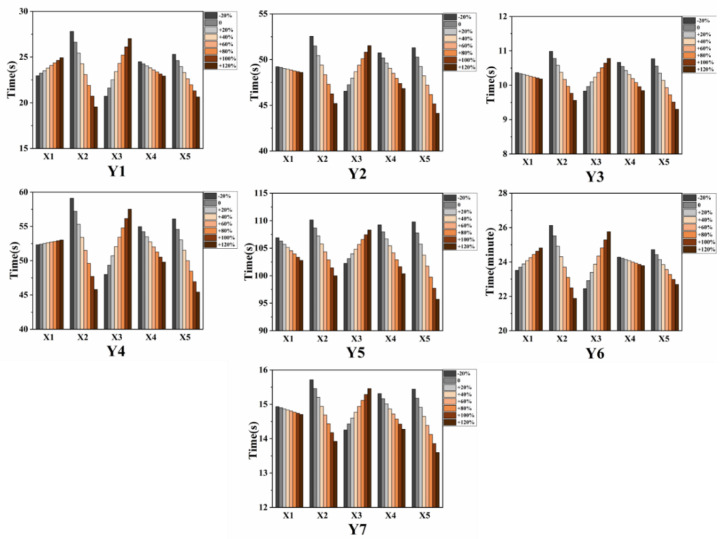
The predicted results of the response variables base on the PLSR model. Y1: the predicted results of the performance of rope climb; Y2: the predicted results of the performance of run 200 m round trip with load; Y3: the predicted results of the performance of run 60 m carrying a ladder; Y4: the predicted results of the performance of climb stairs with a load. Y5: the predicted results of the performance of evacuation of 400 m with supplies; Y6: the predicted results of the performance of run 5 km with an air respirator; Y7: the predicted results of the performance of run 100 m with the water hose.

PLSR models for firefighters from southeast China (Figure 1) were trained separately for the rope climb (Y_1_), run 200 m round trip with load (Y_2_), run 60 m carrying a ladder (Y_3_), climb stairs with load (Y_4_), evacuation of 400 m with supplies (Y_5_), run 5 km with an air respirator (Y_6_) and run 100 m with water hose (Y_7_) tasks. A ‘leave-one-out’ analysis showed a response variables prediction accuracy of 98.79% for the training set and 98.75% for the test set. The results of the sensitivity analysis of the PLSR model based on the independent variable set disturbance factor are shown in Figure 2.

Under the control of other independent variable sets we can find (1) with increased weight, under the same conditions, the time required for firefighters to complete the skills Y_2_, Y_3_, Y_5_ and Y_7_ will be reduced, and the time required for firefighters to complete the skills Y_1_, Y_4_ and Y_6_ will be increased, but there is little change in the time required to complete special skills Y_2_, Y_3_ and Y_4_. (2) With the gradual increase in VO_2max_, the time required for a firefighter to complete a specific skill under the same conditions showed a significant downward trend. (3) With the gradual increase in body fat percentage, the time required for firefighters to complete specific skills under the same conditions showed a significant increase. (4) With the gradual increase in the peak power of the firefighter’s upper body, the time required for firefighters to complete specific skills under the same conditions showed a significant decrease, but it had little effect in Y_4_ and Y_6_ tasks. (5) With the gradual increase in the peak power of the firefighter’s lower body, the time required for firefighters to complete specific skills under the same conditions showed a significant decrease.

## 4. Discussion

The primary purpose of this study was to identify the relationships between the fitness parameters and various training performances of firefighters on the specific ability tests, which comprised a set of seven simulated firefighting tasks. The overall magnitude of the correlations identified in this study indicates that, regarding the ability tasks performed, front-line firefighters demand appropriate aerobic capacity, body fat percentage and muscular power.

As weight increased, the time required for a firefighter to perform the ability tests under the same conditions was inconsistent, and body fat percentage may be a better fitness index. We chose body fat percentage as one of the fitness parameters because it is more closely related to many physical fitness factors [33]; body mass index (BMI) is related to fat mass, as well as fat-free mass, but its application in exercise populations has been questioned [34]. Obesity based on BMI classifies people with lower weight despite excessive fat as normal, such as the elderly, and classifies young people with less fat and more muscle as obese [35]. Our study demonstrated that high body fat percentage was associated with poor test performance on each of the seven ability tasks. The correlation between body fat percentage and ability of firefighters was in agreement with previous studies [36,37,38,39] that demonstrated positive relationships between body fat percentage and each task performance time, which supports the concept that a high body fat percentage is related to poor test performance on simulated firefighting ability tasks. Davis et al. [40] suggested that excess body fat puts an extra burden on the musculoskeletal and cardiovascular system, as it might play a large role in the decline of firefighters’ performance. Therefore, keeping an appropriate body fat percentage seems to be an essential component in ensuring the quality of firefighter performance.

Firefighting requires high levels of aerobic fitness. Various aerobic fitness values reported in the literature are based on submaximal exercise tests, and the reported aerobic fitness values for firefighters are between 35 and 56 mL/kg/min [4]. Based on research results, the National Fire Protection Association (NFPA) Standard on Occupational Medical Programs for Fire Departments recommends that firefighters have a minimum aerobic capacity of 42 mL/kg/min [41]. Our study also has shown that maximal oxygen uptake (VO_2max_) was significantly related to firefighting ability tests. Storer et al. [8] suggested that low aerobic capacity is often insufficient to safely and effectively meet the physiological needs of firefighters for high-intensity work rates. Aerobic capacity is an important factor in the performance of many firefighting tasks, especially those involving firefighting. As firefighters must be able to maintain a relatively intense submaximal workload for numerous minutes at a time, high-level aerobic fitness is indispensable [5]. Despite this, a few studies [17,18,19] suggested that performance on a general aerobic fitness test is only moderately related to performance in rescue operations or fire suppression activities. Given the physical requirements of firefighting, and the high proportion of deaths due to cardiac events during duty [42,43], it is essential for firefighters to take endurance training.

Muscular power is the product of force and speed of muscle contraction. Muscular power is important to meet the physical demands of firefighters as it is necessary for them to complete the tasks [4]. The results of this study demonstrated that the muscular power of the upper body and muscular power of the lower body were both inversely related to each test completion time. The negative correlations indicated that as muscular power or strength increased, performance speed on each of the ability tests improved, showing the importance of power or strength in completing the tests on time. The correlation between muscular power or strength and ability of firefighters were in agreement with previous studies [36,38]. Gledhill et al. [44] described that during emergencies, firefighters may carry various weights, in addition to the extra weight they usually carry (protective clothing and self-contained breathing apparatus weight). Firefighters also need to constantly work against the pressure of charged hoses [37]. The nature of firefighting may explain the correlations between muscular power and ability tasks, due to many tasks (forcible entries, chopping tasks, pulling hoses, lifting and carrying or dragging victims, and carrying heavy equipment and hoses) requiring high levels of muscular power to perform.

Firefighting is a job that requires an extremely physically demanding and high level of fitness to perform safely and adequately. A better understanding of the specific fitness parameters associated with increased or decreased firefighting performance would allow firefighters and instructors to fully prepare for the physical part of the job [37]. During fire academy training, the firefighters may be educated about the benefits of physical conditioning programs on task performance [45]. In this study, PLSR was used to identify a subset of fitness parameters that could predict time on completing each of seven tasks, and the results have shown that predictor variables were suitable predictors of all seven simulated firefighting task scores. One of the most desirable outcomes of this study is the specific variables that would predict high-level firefighting performance. Finding correlations and contributions of individual variables with the ability to perform firefighting tasks would enable firefighters to focus on the effects of physical condition as well as help instructors to select or design appropriate protocols and measures to test the performance of firefighters.

However, some limitations should be considered. Firstly, the subjects of this study were all male firefighters, and the data set did not include female test records to conduct a similar analysis. Secondly, firefighters did not dress in protective clothing and carry self-contained breathing apparatus during the ability tests, which could make the test less demanding than actual firefighting. Lastly, the sample size was limited to a relatively small municipal fire department with only 20 firefighters, and they have not been representative of all firefighters in South China.

## 5. Conclusions

The present study demonstrated significant relationships among physical health parameters and performance on simulated firefighting ability tasks. As described in all seven tasks, high levels of aerobic capacity, low body fat percentage and high levels of both upper body muscular power and lower body muscular power were significantly associated with improved task performances, which also represent that those parameters contributed significantly to the model’s predictive power and were suitable predictors of simulated firefighting task scores. The correlations and contributions of individual physical health parameters with the ability to perform firefighting tasks would enable firefighters to focus on the effects of specific physical conditions during their physical training programs, and it would also encourage instructors to select or design a complete assessment of physical fitness for firefighters.

## Figures and Tables

**Table 1:** The average value (X_ave_), maximum value (X_max_), minimum value (X_min_) and the difference between the maximum and minimum values (X_dif_) of predictive variables X.

X	X_1_ (kg)	X_2_ (mL/kg/min)	X_3_ (%)	X_4_ (W)	X_5_ (W)
X_ave_	69	46.85	14.655	675.35	1705
X_max_	90	62	22.2	921	2564
X_min_	56	29	7.7	483	1408
X_dif_	34	33	14.5	438	1156

X: predictor variables, X_1_: weight, X_2_: maximum oxygen uptake, X_3_: body fat percentage, X_4_: upper body muscular power, X_5_: lower body muscular power, W: Watt.

**Table 2:** The predictors of each predictive variable.

X	X_1_ (kg)	X_2_ (mL/kg/min)	X_3_ (%)	X_4_ (W)	X_5_ (W)
X_min_ − 20% X_dif_	49.2	22.4	4.8	395.4	1176.8
X_min_	56	29	7.7	483	1408
X_min_ + 20% X_dif_	62.8	35.6	10.6	570.6	1639.2
X_min_ + 40% X_dif_	69.6	42.2	13.5	658.2	1870.4
X_min_ + 60% X_dif_	76.4	48.8	16.4	745.8	2101.6
X_min_ + 80% X_dif_	83.2	55.4	19.3	833.4	2332.8
X_max_	90	62	22.2	921	2564
X_min_ + 120% X_dif_	96.8	68.6	25.1	1008.6	2795.2

X: predictor variables, X_1_: weight, X_2_: maximum oxygen uptake, X_3_: body fat percentage, X_4_: upper body muscular power, X_5_: lower body muscular power, W: Watt.
